# Adolescent road safety: pedestrian behavior in ADHD and typically developing groups

**DOI:** 10.1186/s40359-025-03704-x

**Published:** 2025-12-09

**Authors:** Elizabeth Doerr, Andrea Baldassa, Agnese Capodieci, Massimiliano Gastaldi, Chiara Meneghetti, Veronica Muffato, Federico Orsini, Riccardo Rossi, Mariaelena Tagliabue, Barbara Carretti

**Affiliations:** 1 Department of General Psychology, University of Padova, Via Venezia, 8, 35131 Padua, Italy; 2 Department of Civil, Architectural and Environmental Engineering, University of Padova, Via Marzolo, 9, Padua, 35131 Italy

**Keywords:** Adolescents, Road, Pedestrians, ADHD, Walking, Crossing, Virtual scenario

## Abstract

**Background:**

Interaction with the road environment as a pedestrian begins early, increasing during adolescence with growing independence. However, pedestrians face crash risks, particularly those with Attention Deficit Hyperactivity Disorder (ADHD).

**Methods:**

This study compared the road-crossing and sidewalk-walking behaviors of adolescents with and without ADHD in virtual road scenarios. Twenty-one participants with ADHD and 21 adolescents with typical development from 11 to 16 years of age, paired for gender and intelligence participated in the study.

**Results:**

Adolescents with ADHD displayed more unintentional risky behaviors, such as wandering while crossing, looking away, and taking longer to avoid obstacles. However, they also showed positive behaviors, such as checking both sides of the road before crossing. Manifestations of inattention, as observed by parents, were associated with these risky behaviors.

**Conclusions:**

Our results extend previous findings, showing that ADHD puts adolescent pedestrians at greater risk in the road context, despite manifesting also potential positive behaviors.

## Introduction

Pedestrians are among the most vulnerable users in the road environment, as they are highly susceptible to injuries in accidents [1] and lack protective equipment to shield them from collisions with larger elements, such as vehicles. While data indicate a downward trend in road traffic mortality among pre-adolescents and adolescents [2], stalled in recent years in high-income countries [3], road traffic injuries remain a leading cause of death in youths [4, 5]. Indeed, younger pedestrians are particularly at risk due to various factors. Some are related to the design of road environments, which are typically tailored to adult characteristics [3], while others pertain to individual factors. For instance, children and adolescents may struggle to recognize dangerous situations or anticipate others' behavior [6]. Research suggests age-related differences within younger groups: children are more likely to face unintentional exposure to road risks, whereas adolescents are prone to engage in risk-taking behaviors [7, 8]. Additionally, certain types of violation, such as crossing during a yellow light, are more common among pedestrians under 30 years of age, with male pedestrians showing a higher tendency to violate such rules [9].

To better understand the factors underlying pedestrian crashes, recent studies advocate distinguishing between deliberate and unintentional (involuntary) risky behaviors [10, 11]. Specifically, intentional risky behaviors, such as crossing when the traffic light is red, are contrasted with errors which are unintentional but still hazardous actions on the road. Unintentional risky behavior refers to actions that do not involve an intention to violate rules but can be attributed to distraction or poor judgment of the context or the consequences of one’s own actions. Examples of such behaviors include stopping or changing direction while walking, or looking at a billboard instead of concentrating on traffic (see, for example, [11] questionnaire). Both intentional and unintentional risky behaviors are recognized as critical concerns for road user safety and well-being (Hezaveh et al., 2018 [12]).

Following this distinction, the present study focuses on behaviors that can be categorized as unintentional risky behaviors in individuals with and without a diagnosis of Attention Deficit Hyperactivity Disorder (ADHD). This focus is motivated by recent findings suggesting that such behaviors are associated with self-reported pedestrian crashes [13]. The literature further indicates that individuals with ADHD often experience difficulties in maintaining attention [14], are easily distracted by irrelevant stimuli [15], struggle with sequencing actions or maintaining rhythm [16], and may have trouble anticipating the consequences of their behavior (e.g., [17]). These characteristics, in turn, increase the likelihood that individuals with ADHD will engage in actions that enhance their risk of injury (e.g., [18]). In the light of these findings, the present study aims at analyzing the road behavior of individuals with and without a diagnosis of ADHD across two different scenarios: a crossing and a walking task. The following paragraph reviews the literature on ADHD and pedestrian behavior, providing the context to which the present study contributes.

### ADHD and pedestrian behavior

ADHD is a neurodevelopmental disorder characterized by a constellation of manifestations which make individuals more prone to injuries [18]. For instance, individuals with ADHD may lose attention during the execution of a task, or may react impulsively to an event, or may pay attention to irrelevant information [19]. A systematic review by Brunkhorst-Kanaan et al. [18] revealed that individuals with ADHD face a growing risk of injuries as they age. The highest number of injuries is observed in the 12–18 and 18–25 age groups. The authors also identified variations in the frequency and types of injuries across different age groups. For instance, poisoning and ingestion-related injuries are more prevalent during childhood, whereas motor vehicle accidents become increasingly common in adulthood. In the context of road safety, children are particularly vulnerable to injuries as pedestrians as demonstrated by several studies [20].

For example, Stavrinos and colleagues (2011) compared pedestrian behavior of 7–9 years old participants with and without ADHD, using a semi-immersive interactive virtual environment, assessing pedestrian behavior in crossing scenarios. They considered different variables related to the initiation of crossing (curbside behavior) and the safety of crossing, i.e. when they decide to cross the road. The results showed that children with ADHD demonstrated adequate curbside pedestrian behavior, comparable to that of their typically developing peers: they waited before crossing, looked both ways, and missed a similar number of safe crossing opportunities. However, once they began to cross, they engaged in riskier behaviors, that is choosing more frequently unsafe locations and less optimal moments to cross the road.

More recently, the systematic review by Wilmut and Purcell [21] confirmed this trend, showing that children and preadolescents with ADHD correctly plan road crossing, for example by looking right and left before crossing, but make poor temporal gap choices, as in shorter temporal gaps for crossing, between oncoming vehicles.

Other evidence was brought by Tabibi and colleagues (2022). They compared children aged between 8–12 years old with and without a diagnosis of ADHD in a virtual road crossing task. In this study, the authors manipulated traffic density, considering three levels of complexity; the results showed that all the children manifested riskier road crossing behaviors with greater traffic density, but the level of risk increased especially in children with ADHD. Indeed, they crossed in an unsafe manner, with poorer temporal gap choices and lower collision time with oncoming vehicles (see also [22]).

A more recent study by Tabibi and colleagues (2023) examined crossing behavior in children with and without ADHD, aged 7–12 years. Participants were presented with VR crossing scenarios, where pedestrian behavior was analyzed in terms of unsafe pedestrian crossing, start delays to enter safe traffic gaps, and attention to traffic. The results showed that children with ADHD exhibited riskier crossing behaviors, while no significant differences were observed between the groups for start delays or attention to traffic. Additionally, the study analyzed associations between these behaviors and parent-reported executive functioning in everyday life tasks. In both groups, a higher frequency of executive function deficits—as reported by parents through a questionnaire (e.g., poor time management and self-organization)—was associated with a greater number of unsafe crossings in the Virtual Reality (VR) scenarios.

These findings are particularly interesting for the present study as they suggest that manifestations of executive control problems are linked to risky pedestrian behaviors independently of the presence of a specific diagnosis.

To summarize, the literature suggests a significantly different pedestrian behavior in individuals with ADHD compared to their peers: while children with ADHD demonstrate adequate curbside behaviors, they often engage in riskier road-crossing behaviors, including poor temporal gap choices and unsafe crossings, particularly under complex traffic conditions.

However, most studies in this area have focused on road crossing, as this activity is associated with a higher incidence of injuries (e.g., [23]). In contrast, basic walking behavior, that is, walking without crossing situations, has been seldom examined in individuals with ADHD. An example being the study by Krasovsky et al. [24] who analyzed gait under a dual-task condition (walking while texting on mobile phone) in individuals with and without ADHD. Although no differences emerged in most of the variables considered, higher symptoms of hyperactivity were associated with a higher dual task cost. Examining other road-related situations, such as walking along the street, may therefore provide valuable insights into whether at-risk behaviors in individuals with ADHD occur exclusively in cognitively demanding contexts—such as road crossing (see, for example, [25])—or whether they also extend to more routine everyday situations, such as walking on the sidewalk.

Furthermore, most of the studies reviewed above involved younger participants, typically between 8 and 12 years of age, whose pedestrian experiences are largely supervised by adults. In contrast, the present study focuses on pre-adolescents, a developmental stage in which individuals begin to move autonomously within road environments. Analyzing their walking and crossing behaviors can therefore shed light on factors that may place them at particular risk during this critical transition toward independent mobility.

#### Aims of the study

Based on previous evidence from the literature, the present study has two main aims.

The first objective (AIM 1) of this work was to better understand differences between adolescents with and without ADHD in common road situations, such as crossing and sidewalk walking. We focused on variables related to behaviors that may unintentionally increase the risk of injury. In particular, we considered variables usually considered in the field, such as walking speed and safe gap selection in the crossing situations [25, 26], as well as wandering while walking, that is, the extent to which individuals deviated from a straight path. Previous research has shown indeed that unpredictable pedestrian behaviors (e.g., hesitation or erratic movement) in road environments can contribute to crashes and should therefore be regarded as risk factors for injury [27].

In addition, we examined other behaviors that may increase accident risk, such as head wandering while walking and delayed reactions to unexpected stimuli, for example, the sudden opening of a parked vehicle’s door.

Finally, and in line with previous literature [21], we also included positive safety behaviors, such as head rotations to the left and right before crossing, which reflect attentional monitoring of the environment.

On the basis of the literature [18], we hypothesize group differences, especially less optimal pedestrian behavior in ADHD, for both crossing and walking scenarios. Different results are present in the literature regarding walking speed, with some studies reporting a slower [25] or faster pace (e.g., [28]) in participants with ADHD, with respect to their peers. It is to note that, when participants with ADHD are instructed to walk as they usually do, no significant differences tend to emerge (see, for instance, [29]), so, no difference between groups might be found in speed.

When considering movement wandering, we expect to find that participants with ADHD do not follow a straight path while walking (movement wandering), due to their symptoms related to inattention and hyperactivity [30], in both crossing and walking scenarios. According to previous studies, we additionally expect to find shorter safe-gaps in participants with ADHD with respect to the typical group in crossing scenarios, but also adequate behavior such as head rotations left and right before crossing [21]. Although no previous evidence is present, we expect greater head wandering and delay to respond to unexpected events during walking, since easy distractibility is a key component of ADHD in adolescence [31].

The second aim (AIM 2) of the study was to explore the relationship between inattention and impulsivity/hyperactivity symptoms (reported by parents) taken as continuous traits, and pedestrian behavior in the two kinds of scenarios. Inattention and impulsivity/hyperactivity are two core dimensions of ADHD, but they are also present in typical populations. Examining the associations between inattention and hyperactivity–impulsivity traits and risky behavior may be important for understanding which of these dimensions has a greater impact on pedestrian behavior. Such analyses may also inform research on other psychopathological conditions characterized by executive dysfunctions (see for example [32]), by clarifying how the presence of these traits can impair behavior in everyday life situations.

On the basis of previous literature, we expect to find a relationship between riskier pedestrian behavior (in crossing and walking scenarios, especially those including more stimuli like incoming vehicles or traffic lights) to inattention and hyperactivity symptoms reported by parents (considered as continuous traits), for both scenarios [33].

## Methods

### Participants

The study involved 42 participants (12 females) from 11 to 16 years of age (M = 12.62, SD = 1.06), 21 (6 females) individuals with Attention Deficit Hyperactivity Disorder (ADHD) and 21 (6 females) with Typical Development (TD), matched for age and gender. For both groups, inclusion criteria were: Italian as a first language; absence of sensory limitations and neurological impairments. In addition, inclusion in the ADHD group required a formal diagnosis of ADHD based on the criteria outlined in the *Diagnostic and Statistical Manual of Mental Disorders-TR, 5th Edition*, (APA, 2022). The diagnosis was made on the basis of a clinical interview and the administration of standardized rating scales filled in by parents and teachers (SDA rating scales, [34]). These scales are commonly used in Italy to make the diagnosis of ADHD,each version is composed of 18 items requiring to report the frequency of inattention (9 items) and hyperactivity/impulsivity (9 items) symptoms. None of the participants with ADHD were under medication treatment. Regarding comorbidities, participants with ASD or oppositional/provocative disorders were excluded, while those with a concurrent diagnosis of specific learning disorder or emotional difficulties were included. Both groups were administered a nonverbal intelligence test [35], a visuo-spatial working memory task – the backward Corsi block task – [36] and a mental rotations task [37] as control variables. As reported in Table 1, the two groups did not differ in any of the above-mentioned variables. In addition, parents and teachers of the children in the control group filled in the same rating scale used for the diagnosis of the ADHD group [34]. As expected, in this case differences emerged with a higher frequency of inattention and hyperactivity/impulsivity symptoms for the group of adolescents with ADHD (Cohen’s *d* = 2.67 for inattention and *d* = 1.82 for hyperactivity/impulsivity). The Ethical Committee for Psychological Research at the University of Padova approved the study (No. 5161). Informed consent was collected before the experiment, with parents (or guardians) providing a signed consent.

**Table 1:** Descriptive statistics of screening measures and F-test values for the two groups (ADHD and TD)

	ADHD group *N* = 21 (6 F)		TD group *N* = 21 (6 F)				
	Mean	sd	Mean	sd	*F* (1, 40)	*p*	*η*^*2*^
Age	12.86	1.24	12.38	0.81	2.19	0.147	0.052
Nonverbal intelligence	28.71	7.01	30.71	5.29	1.09	0.303	−0.027
Visuo-spatial working memory	4.91	1.14	5.29	1.23	1.09	0.303	0.026
Mental rotation	3.76	2.49	4.00	2.39	0.10	0.753	0.002
**Inattention**	**16.60**	**5.78**	**3.95**	**2.20**	**87.87**	** <.001**	**0.687**
**Hyperactivity/impulsivity**	**12.27**	**5.48**	**3.81**	**2.54**	**41.15**	** <.001**	**0.507**

### Materials

#### Road behavior simulator

**Fig. 1:**
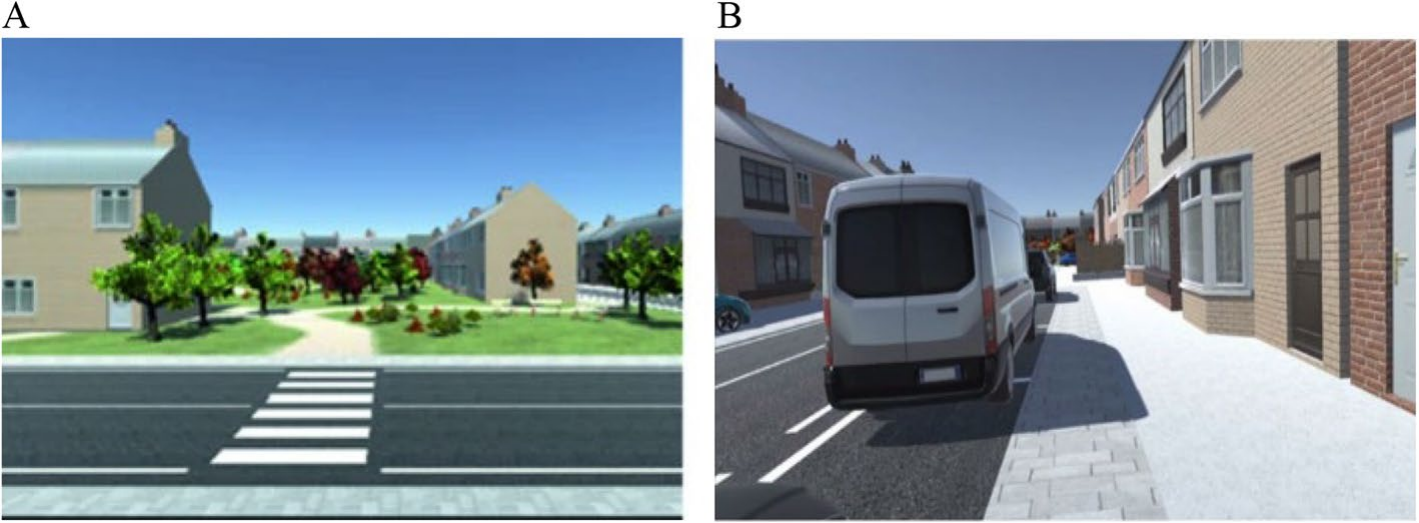
Example of crossing (**A**) and walking scenario (**B**)

A pedestrian simulator, developed by the Transportation Laboratory from the University of Padova [38] was devised to test both road crossing and walking scenarios (see Fig. 1. The simulator is equipped with a backpack PC (HP Backpack VR G2, a Reverb Headset (HP Reverb VR Headset G2 and two joysticks, to allow an immersive free roaming virtual reality experience. Each eye was presented with 2160 × 2160 LCD screens, delivering high resolution and vibrant colors; the refresh rate was 90 Hz. The headset offered a horizontal field of view of 98 degrees and included Valve speakers positioned 10 mm from the ears. The system ensured low latency, contributing to a smooth and responsive virtual experience. During the tests, the backpack PC was wirelessly connected to a monitor, enabling operators to view the same images displayed on the headset in real-time to monitor participants' progress and detect any issues. The scenarios, developed using Unity Software® (2020.3 version, represent typical elements of the road environment (e.g. vehicles, signs, buildings, traffic lights and crossroads and were recently validated for use with adolescents (for further details see [26]). The task includes 2 training sessions (one for crossing situations, the other for sidewalk walking), to allow the participant to gain familiarity with the virtual reality features. The experimental task is composed of 8 crossing scenarios and 5 walking scenarios. Each scenario includes specific elements from the road environment (listed in Table 2).

**Table 2:** Features of each crossing and walking scenario

Crossing scenarios				
0 – training				
1 - no traffic light, no cars				
2* - no traffic light, no road markings, cars coming from the right				
3* - no traffic light, no road markings, cars coming from both sides				
4 - red traffic light, no cars				
5* - red traffic light, with cars				
6 - green traffic light, change to yellow when crossing starts; no cars				
7* - no traffic light, no road markings, cars coming from the right, with distractor horn				
8* - red traffic light, with cars, with distractor horn				
Walking scenarios				
0 – training				
1 - filler (no events)				
2 - parked car opening passenger door				
3 - e-scooter coming out from behind a van				
4 - parked car opening passenger door, with distractor horn				
5 - e-scooter coming out from behind a van, with distractor horn				

*N.B.* Crossing scenarios with

^*^are those used to compute the safe gap variable

For both road crossing and walking scenarios we considered a series of dependent variables. In road crossing scenarios, we measured speed (kilometers per hour). Another variable collected was safe gap as an indicator of whether the participant crossed the road within a safe amount of time between incoming vehicles. This variable was computed only when cars were present in the scenario, and one point was assigned when this behavior occurred. To describe crossing behavior, we computed movement wandering, i.e. how often the person wanders while walking. This variable was computed as the standard deviation of the lateral position while crossing. A higher value indicates greater irregularity in the trajectory followed. We also considered looking both sides as an indicator of whether the participant checked both sides of the road before crossing. In this case, one point was assigned when this behavior was observed.

In sidewalk walking scenarios, in addition to speed (kilometers per hour) and movement wandering, we also considered the percentage of total time that the head was rotated left or right (horizontal axis) and upward or downward (vertical axis) while walking. To compute these two variables, head rotations were tracked. A head rotation event was triggered when the rotation around the vertical axis exceeded 45 degrees, given that the VR headset’s fixed horizontal field of view is 98 degrees (for horizontal head movement). Similarly, for vertical head movement,, an event was triggered when the rotation around the horizontal axis exceeded 45 degrees, considering that the device’s vertical field of view is approximately comparable to the horizontal one.

Considering that in the walking scenario some obstacles were present, we also computed the delay between door opening and the instant the participant moved to the side to overcome the obstacle (sudden opening of a parked car's door). This variable allowed us to measure the time required by each participant to react to the door opening, consisting in speed reduction or trajectory change.

The mean values for each dependent variable across crossing scenarios and walking scenarios was calculated.

### Procedure

Participants of the control group were recruited from local schools, whereas those from the ADHD group came from clinical centers. Parents signed the consent form. Firstly, participants completed the control tasks: the non-verbal intelligence, the mental rotation and the visuo-spatial working memory tasks.

As soon as they finished these tasks, participants were introduced to the walking simulator. The task was carried out in an open space to allow free movement safely, such as a school or local gym. Moreover, a professional was always present to guide and assist the participant. The task was administered individually with each participant. The scenarios were presented in a random order and participants would initiate the task as soon as they received a signal message. They were instructed to behave as they would in the real environment, therefore they were neither encouraged to respect the road rules, nor to ignore them. The administration process lasted about 45 min for each participant (10–15 min for the task with the simulator).

## Results

Groups were compared on the variables of interest with a series of univariate ANOVA (see Table 3 for the results), distinguishing between the type of scenario (crossing or walking). Cohen’s *d* was calculated as a further measure of the significant comparisons (considering the general guidelines for effect interpretation: *d* between 0.2 and 0.5 is a small effect, *d* between 0.5 and 0.8 is medium and *d* higher than 0.8 is a large effect).

**Table 3:** Descriptive statistics and results of the comparison between the ADHD and TD group. Effect size of the differences in the crossing and walking scenarios are also reported. Significant comparisons are highlighted in bold

	ADHD group		TD group						
	Mean	SD	Mean	SD	F (1, 40)	p	η^2^	d	SE of d
Crossing scenarios									
Speed	4.82	1.01	4.80	0.83	0.004	0.948	0.0001	0.02	0.31
**Movement wandering**	**0.14**	**0.05**	**0.10**	**0.03**	**10.65**	**0.002**	**0.210**	**0.97**	**0.33**
**Looking both sides**	**0.52**	**0.21**	**0.35**	**0.25**	**5.78**	**0.021**	**0.126**	**0.74**	**0.32**
Safe gap	0.67	0.38	0.75	0.39	0.68	0.413	0.017	−0.21	0.31
Walking scenarios									
Speed	3.73	0.48	3.69	0.36	0.12	0.728	0.003	0.09	0.31
**Movement wandering**	**0.20**	**0.07**	**0.15**	**0.08**	**5.06**	**0.030**	**0.112**	**0.67**	**.32**
**Head wandering horizontal (percentage)**	**9.87**	**8.25**	**4.21**	**5.03**	**7.20**	**0.001**	**0.153**	**0.83**	**0.32**
**Head wandering vertical (percentage)**	**2.98**	**4.78**	**0.61**	**0.96**	**4.98**	**0.031**	**0.111**	**0.69**	**0.32**
**Lateral door delay**	**3.50**	**1.11**	**2.68**	**0.52**	**9.45**	**0.004**	**0.168**	**0.95**	**0.32**

*SE* Standard Error

### Group differences of ADHD and TD in walking and crossing scenarios (AIM 1)

#### Crossing scenarios

There were no differences between the two groups in speed; however, differences emerged in movement of wandering (*d* = 0.97, large effect size): the group with a diagnosis of ADHD did not follow a straight path during crossing scenarios. Moreover, it should be noted that participants with ADHD exhibited the appropriate behavior of looking left and right before crossing the road (*d* = 0.74, medium effect size) more frequently than their peers. There were no group differences in the safe gap variable (see Table 3).

### Walking scenarios

There were no differences between the two groups in speed (Table 3); however, differences emerged with movement of wandering. In particular, there were group differences in the percentage of head wandering in vertical (*d* = 0.83, large effect size) and horizontal line (*d* = 0.67, medium effect size): participants with ADHD kept more often their head rotated elsewhere when walking. Furthermore, the two groups differed in lateral door delay (*d* = 0.95, large effect size) where participants with ADHD took more time to prepare themselves to avoid an incoming obstacle, such as door opening. In addition, the ADHD group did not follow a straight path while walking (*d* = 0.69, medium effect size) when compared to the control group.

### Correlations of inattention and impulsivity/hyperactivity symptoms with crossing and walking scenarios variables (AIM 2)

Correlations were run considering the whole sample to analyse the relationship of Inattention and Hyperactivity/impulsivity with pedestrian behavior in crossing and walking scenario variables (Table 4).

**Table 4:** Pearson correlations between symptoms of inattention and hyperactivity/impulsivity and mean scores of the variables computed in the crossing and walking scenarios

	Inattentive symptoms	Hyperactivity/impulsivity symptoms
Crossing scenarios		
Speed	0.023	−0.025
Movement wandering	0.557***	0.657***
Looking both sides	0.411**	0.413**
Safe gap	−0.106	−0.119
Walking scenarios		
Speed	0.105	0.108
Movement wandering	0.407**	0.425**
Head wandering horizontal (percentage)	0.280	0.363*
Head wandering vertical (percentage)	0.263	0.385*
Lateral door delay	0.360*	0.312*

^*^p <.05, ** *p* <.01, *** *p* <.001

Significant correlations were found when considering the scores from the Inattention and hyperactivity/impulsivity questionnaire. Specifically, movement wandering during crossing correlated positively with inattention and hyperactivity/impulsivity. A greater tendency of looking both sides before crossing was related with inattention and hyperactivity/impulsivity. Movement wandering when walking shared significant correlations with inattention and hyperactivity/impulsivity. Lateral door delay correlated positively with inattention and hyperactivity/impulsivity. Hyperactivity/impulsivity correlated significantly with head wandering horizontal and vertical.

## Discussion

A growing number of studies is analyzing the behavior of individuals with ADHD as pedestrians. Pedestrians are considered as some of the most vulnerable users in the road environment and certain pedestrians, such as individuals with ADHD, are even more at risk of being involved in road accidents. For example, individuals with ADHD tend to cross in more dangerous parts of the road and select riskier temporal gaps [21, 25, 30]. However, they do not exhibit only risky behaviors,some studies have indeed shown that they are also able to plan their crossings [30] and perform head rotations to the left and right before crossing [21]. As noted in the introduction, while crossing behaviors have been relatively well studied, general walking behavior—that is, walking outside of crossing situations— remains underexplored in the ADHD literature.

Based on these findings, the main aim of the present study was twofold: first, to compare pedestrian behaviors in adolescents with and without a diagnosis of ADHD (AIM 1); and second, to examine the relationship between these behaviors and ADHD-related symptoms from a dimensional perspective (AIM 2).

To tackle these goals, we used outdoor city crossing and walking VR scenarios recently implemented and validated for adolescents (see [26]). Each scenario included typical elements of the road environment, such as traffic lights, road markings or incoming vehicles. Pedestrian behavior was analyzed with measures of involuntary risky behaviors that were considered in line with the literature that associates them to road injuries [13]. In particular, we focused on multiple variables at the same time, such as speed, safe gap choice, regularity of walking (movement wandering) and attention to road environment and risks in two types of scenarios: crossing and sidewalk walking scenarios.

The results have shown some group differences in both crossing and walking scenarios. In the context of crossing scenarios, unlike previous studies, no differences were identified in walking speed between the two groups (e.g. [25, 28]). Adolescents with ADHD and those with TD completed the crossing in comparable times. As already found in the literature, it is noteworthy that walking speed in individuals with ADHD varies depending on the context and the specific task being measured, with some studies reporting slower speed [25] and others faster paces (e.g., [28]). However, when participants with ADHD are instructed to walk as they usually do, no significant differences would emerge (see, for instance, [29]). In our study, the emphasis on behaving naturally may have explain the absence of differences between groups.

Another feature of our scenarios may help explain the absence of group differences in walking speed as well as in the safe gap variable, which contrasts with previous literature [30]. The crossing scenarios incorporated various elements—such as the presence or absence of zebra crossings and traffic lights—that introduced differences in complexity, which could influence participants’ behavior. For example, Tabibi et al. [25] found that risky behaviors were more pronounced in complex road environments, with larger differences between individuals with ADHD and control participants. Future studies should therefore operationalize scenario characteristics more precisely to clarify their impact on appropriate street-crossing decisions.

Group differences, however, emerged in other variables considered in the study: participants with ADHD wandered more frequently than their peers. This finding seems interesting as deviations from a straight path may increase the risk of accidents, due to the unpredictability of pedestrian behavior [39].

In addition to these potentially risky behaviors, individuals with ADHD also demonstrated a seemingly appropriate behavior by looking both sides before crossing—a pattern previously reported in the literature [21]. However, the current study does not evaluate whether this behavior resulted in more effective hazard detection or safer crossing choices. This represents a limitation, as prior research suggests that such looking behavior may not always reflect actual situational awareness. For instance, Stavrinos et al. [30] found that participants with ADHD exhibited head movements consistent with scanning behavior (i.e., turning left and right), nonetheless their crossing behavior resulted in a higher frequency of unsafe crossings compared to a control group. These findings suggest that individuals with ADHD may "look but not see" relevant information. This may be particularly relevant considering common daily life situations in which participants are occupied in a second task, for example cell phone texting. In their meta-analysis, Stavrinos et al. [40] summarized the negative impact of mobile phone use on both pedestrian and driver safety. Considering the case of pedestrians, they reported that studies on distracted walking, using both virtual reality and real-world observations, consistently show that mobile technology use negatively affects pedestrian safety across age groups. Visually demanding distractions, such as texting, lead to longer waiting times, slower crossing speeds, missed safe-crossing opportunities, and altered gait patterns. Even considering not visual distractions, talking on the phone, result in delayed crossing and reduced safety. Similar patterns have been observed in the driving literature, where individuals distracted by tasks such as cell phone conversations appear to engage in normal scanning behavior but still fail to register critical visual cues (e.g., [41, 42]). Although not specifically analyzed in our study, these findings underscore the importance of examining potentially risky behaviors even in simple walking conditions that do not involve dual tasks, particularly in individuals with ADHD, who are more susceptible to distraction by irrelevant environmental information.

In the walking scenarios, again no differences emerged with speed, whereas it emerged that participants from the ADHD group wandered more often when walking and kept their head rotated elsewhere. Furthermore, they took more time to prepare themselves for an incoming obstacle (e.g., a door opening).

Overall, the comparison between the groups highlights a number of variables that may help to understand the causes of road accidents in people with ADHD, including the unpredictability of their behavior when walking, which is reflected in changes in walking direction, and their susceptibility to distraction by environmental information, as suggested by the results on head rotation.

Groups differences and in particular the association with ADHD symptomatology can be better understood examining the relationship between these variables and symptoms associated with ADHD, such as inattention and hyperactivity/impulsivity (AIM 2). It emerged that greater symptoms of inattention were associated with wandering when walking (in both crossing and walking scenarios), looking both sides before crossing and taking more time to avoid a door opening from a car parked nearby. Participants with greater hyperactivity/impulsivity wandered more when walking (in both crossing and walking scenarios), looked both sides before crossing, kept their head rotated elsewhere when walking and took more time to avoid the door opening. These results extend current literature (e.g. Falembam et al., 2024) by showing that the intensity of ADHD symptoms, independently from the diagnosis are associated to potentially risky behavior. It would be interesting, in future research, to ascertain if the differences observed between symptoms of inattention and hyperactivity/impulsivity are confirmed in larger samples. Moreover, the role of executive functions (not assessed in the present study) in accounting for these differences should be investigated, especially with regard to executive control that has been proved to be associated with riskier behaviors in ADHD [43].

## Limitations

Although the potential relevance of these findings, some limitations must be acknowledged. Firstly, even though the ADHD sample size is in line with the research in the clinical domain, it is still relatively small and this limits generalizability of the results found and reduces the statistical power to detect smaller effects. Future studies should for example examine additional individual factors, such as socio-economic status and comorbidities, which may influence outcomes. Moreover, gender differences warrant attention, as prior research (e.g., Wang et al., 2018) has shown that girls tend to adopt safer crossing behaviors with age, while boys often continue to engage in riskier behaviors. Although the prevalence of male is higher in the group of individuals with a diagnosis of ADHD, considering also this variable can be relevant to elaborate specific prevention plans.

As already mentioned, an aspect that warrants further exploration concerns the extent to which the behaviors considered in the present research are truly unintentional. In categorizing them as unintentional, we followed the proposal of Useche and Llamazares [13]. However, it should be noted that we did not ask participants about their intention to engage in such behaviors. For this reason, we cannot rule out the possibility that participants were aware or not of performing something dangerous. This issue warrants further investigation.

Another limitation regards the experimental apparatus. While VR offers a controlled and safe setting to investigate complex behaviors, it also presents limitations in terms of ecological validity. In particular, participants were aware that they were in a simulated, low-risk environment, which might have influenced their decision-making and reduced their perceived consequences of unsafe actions. This awareness could lead to behaviors that differ from those exhibited in real-life traffic situations, potentially limiting the generalizability of the findings to actual pedestrian safety and risk assessment. Lastly, a further limitation concerns the use of scenarios that differed in the number and type of road elements, such as traffic lights and parked vehicles—factors that can influence perceived complexity and, consequently, participants’ interactions with the road environment (see [25]).

## Conclusion

To conclude, pedestrian behavior, especially among adolescents who are beginning to navigate road environments independently, represents a critical area of research. Our study highlights that preadolescents with ADHD exhibit several involuntary risky behaviors when faced with road-crossing and walking scenarios. Notably, inattention appears to play a significant role in these behaviors, potentially increasing the vulnerability of road users in real-world situations. From an applied perspective, the current results, if replicated, may help inform the development of interventions for adolescents with ADHD. For example, such interventions could focus on increasing awareness of how they interact with the road environment, reflecting on the attention paid to traffic signals, and promoting more predictable walking behaviors. These interventions can help individuals with ADHD in developing safer pedestrian habits, ultimately reducing risks and enhancing their autonomy in everyday environments. 

## Data Availability

The dataset analyzed in the current study is available on Figshare at the following DOI: 10.6084/m9.figshare.28959368.

## Abbreviations

ADHD: Attention Deficit Hyperactivity Disorder
VR: Virtual reality
TD: Typical Development
